# Comparative phylogenies and host specialization in the alder ectomycorrhizal fungi *Alnicola*, *Alpova *and *Lactarius *(Basidiomycota) in Europe

**DOI:** 10.1186/1471-2148-11-40

**Published:** 2011-02-09

**Authors:** Juliette Rochet, Pierre-Arthur Moreau, Sophie Manzi, Monique Gardes

**Affiliations:** 1 Université de Toulouse, UPS, UMR 5174 EDB (Laboratoire Evolution et Diversité Biologique), 118 route de Narbonne, 31062 Toulouse Cedex 4, France; 2 CNRS, UMR 5174 EDB, 31062 Toulouse Cedex 4, France; 3 Laboratoire de Botanique, Faculté des Sciences pharmaceutiques et biologiques, Univ Lille Nord de France, 59006 Lille Cedex, France

## Abstract

**Background:**

Mycorrhizal fungi form intimate associations with their host plants that constitute their carbon resource and habitat. *Alnus *spp. (Betulaceae) are known to host an exceptional species-poor and specialized ectomycorrhizal (ECM) fungal community compared to other tree species, but the host-specificity pattern and its significance in terms of fungal diversification and speciation remain poorly documented. The degree of parallel speciation, host switching, and patterns of biogeography were explored in the historical associations between alders and three ECM taxa of Basidiomycetes: *Alnicola *(Agaricales), *Alpova *(Boletales), and *Lactarius *(Russulales). The aim was to develop an evolutionary framework on host specificity and diversification of Basidiomycetes in this highly specialized plant-fungus symbiosis.

**Results:**

Sporocarps of *Alnicola *(220), *Lactarius *(61) and *Alpova *(29) were collected from stands of the four European alder species (*A. alnobetula *including the endemic subsp. *suaveolens *in Corsica, *A. cordata*, *A. glutinosa*, *A. incana*) in Western Europe (mainly in France and Austria), from 1995 to 2009. Specimens were morphologically identified to the species level. From these, 402 sequences of four DNA regions (ITS, rpb2, gpd, and the V9 domain of the mit-SSU rDNA) were successfully obtained and analyzed in addition with 89 sequences available in GenBank and UNITE databases. Phylogenetic analyses were conducted on all sequence data sets (individual and combined) using maximum likelihood reconstruction and Bayesian inference. Fungal phylogenies are compared and discussed in relation to the host, with a focus on species boundaries by associating taxonomic, systematic and molecular information.

**Conclusions:**

Patterns of host specificity and phylogenies of *Alnicola *and *Lactarius *suggest coevolution as a basal factor of speciation in relation with the subgeneric diversification of *Alnus*, possibly due to the very selective pressure of the host. A second element of the historical associations between *Alnus *and its fungal symbionts is a host-dependent speciation (radiation without host change), here observed in *Alnicola *and *Alpova *in relation with *Alnus *subgen. *Alnus*. Finally host shifts from *Alnus *subgen. *Alnus *to *A. alnobetula *are found in most lineages of *Alnicola *(at least four times), *Alpova *(twice) and *Lactarius *(once), but they do not represent such a common event as could be expected by geographic proximity of trees from the two subgenera. However, active or very recent host extensions clearly occurred in Corsica, where some fungi usually associated with *Alnus glutinosa *on mainland Europe locally extend there to *A. alnobetula *subsp. *suaveolens *without significant genetic or morphological deviation.

## Background

In the ectomycorrhizal (ECM) symbiosis, the root system of an individual tree is typically colonized by several members of different ECM fungal species, and individual fungi can normally associate with several plants [1]. Ectomycorrhizal fungi display a great range of host specificity [2,3]. This varies from extremes such as *Suilloideae *that are almost exclusively associated with *Pinaceae *[4] with the example of the very host-specific *Suillus pungens *colonizing one or few related pine species [5], to species as *Laccaria amethystina *that demonstrate a true multihost ability [3]. The tight affinity between many ECM fungal species and their hosts has led to host-based taxonomic treatments in certain genera, *e.g. Leccinum *[6], and implicitly suggests that the phylogeny of the fungi follows that of their host plants, a process commonly known as coevolution [7]. Two main categories of events can be proposed to explain highly specialized associations between a plant and a fungal symbiont: (i) cospeciation/codivergence events where a symbiont speciates in response to the speciation of its host (association by descent), the phylogenetic outcome is congruent phylogenies of interacting taxa, and (ii) speciation through host shifts where the symbiont switches from the ancestral host to a new, unrelated host species (association by colonization); the phylogeny of the symbiont is influenced by the host evolution, but it is not reciprocal. Both processes suggest that host diversity, if not necessarily the sole cause of reproductive isolation and speciation, may force diversification and speciation of their symbionts. Host specificity is generally expressed as a symbiont's adaptation to a particular host species or higher taxa. The model investigated in the present study is the ectomycorrhizal symbiosis between the alder genus *Alnus *Mill. (Betulaceae) and three genera of Basidiomycota.

Based on fossil pollen evidence, the plant genus *Alnus *would have originated from tropical Eastern Asia around the Late Cretaceous [8], and it is likely that it reached Europe during early Oligocene. It was diversified in central Europe tropical forests by the end of Oligocene - early Miocene [9], and adapted to temperate and subarctic environments at the Miocene period [8]. Species of *Alnus *are now distributed in temperate and arctic regions of the Northern Hemisphere, except *A. acuminata *Kunth (*sensu lato*, including *A. jorullensis *Kunth) that extends as far South as South America. There are 29-35 species of *Alnus *is the current flora, with 4-5 species in Europe, 9 in the New World, and 18-23 in Asia [8,10,11]. *Alnus *consists of three clades [11,12] including one subgenus not represented in Europe (subgen. *Clethropsis*), and two widely distributed subgenera *Alnobetula *and *Alnus*. The subgenus *Alnobetula *(also described as genus *Duschekia *Opiz) is sister to subgenera *Clethropsis *and *Alnus*, and likely the most primitive one [11-13]. It is represented, according to authors, either by a single thicket-forming circumpolar species: *Alnus alnobetula *(Ehrh.) K.Koch [also known as *A. viridis *(Chaix) DC.] divided into geographical subspecies [8,10], or by several allopatric or parapatric species [11,12]); *A. alnobetula *was already present in France at the late Miocene, about 5.34 My ago [14]. The second main lineage (subgenus *Alnus*) is represented by numerous species in Eastern and Central Asia, with radiations towards North and South America, Europe, the Mediterranean basin and Eastern Asia, differentiated during Pleistocene. In subgen. *Alnus*, *A. glutinosa *(L.) Gaertn is a European and North African endemic species, present from W Europe and N Maghreb to Fennoscandia; pollen records and molecular data have revealed distinct major southern refuges in the last ice age, including W France, Corsica, S Italy, N Africa, Carpathians, and Turkey [15,16]. *Alnus incana *(L.) Moench is distributed across the cooler parts of Europe, mainly in Northern Europe and high elevation mountains in the Alps, the Carpathians and the Caucasus. *Alnus cordata *(Loisel) Duby is a Tyrrhenian endemic species that was isolated during the Pleistocene in Corsica and in a few other Mediterranean ice-free areas (Southern Italy, Albany) [17,18] where it remained confined since its massive introduction for forestry all over Europe during the late 20^th ^century.

After the Quaternary glaciations the circumpolar species *Alnus alnobetula *and *A. incana *expanded in continental Europe (throughout Alps and Carpathians for *A. alnobetula*, up to Eastern France and Scandinavia for *A. incana*) from several refugia located in Central and Eastern Europe [10,19], while *A. alnobetula *subsp. *suaveolens *(Req.) Lambinon & Kerguélen likely evolved isolated as an endemic subspecies in Corsica since the Pleistocene [20,21].

From analysis of mycorrhizae [22-27] alder trees have revealed an exceptional species-poor assemblage of ECM fungi compared to the other tree species, with less than 50 fungal species (including unidentified taxa) reported worldwide in the literature on *A. acuminata*, *A. alnobetula *s. lat., *A. glutinosa*, *A. incana *s. lat., and *A. rubra*. The fungal communities are dominated by six Basidiomycete genera, whatever the species of *Alnus *considered: *Tomentella *(12-15 spp.), *Alnicola *(15-20 spp.), *Lactarius *(5-8 spp.), *Cortinarius *(6-10 spp.), *Alpova*/*Melanogaster *[28] (6 spp.), and *Russula *(2-4 spp.). The fruiting community is also composed of two locally abundant taxa, *Paxillus *(1-2 spp.) and *Gyrodon *(1 sp.), rarely found from mycorrhizae analysis (Rochet *et al*., unpublished results). Other occasionally reported genera are *Amanita *(*A. friabilis*), *Hebeloma, Inocybe*, *Pachyphloeus*, *Pseudotomentella*, and unidentified Helotiales (possibly root endophytes). The *Alnus*-ECM fungi association is considered the most specialized ECM symbiosis. Except *Tomentella *spp. (for which precise taxonomic information is lacking) and several species of Ascomycota [26], all species are known, or strongly suspected, to be exclusive to the genus *Alnus *since they have never been found on any other trees than alders at present. There are reports of species-poor and specialized ECM fungal community for the ectomycorrhizal larch tree *Larix *spp. and five-needle pines (*e.g. Pinus cembra*, *P. strobus*) [29-31]. High specificity patterns are also observed for many orchids and monotrope plants associated with ectomycorrhizal fungi [1].

For the mycorrhizal symbiosis, it is still an open question how associations between plants and fungi arise and how specificity occurs. One way would be geographical isolation of populations leading to a narrowing of host range and allopatric speciation. However, we hypothesize than in highly specialized associations such as the alder-ECM symbiosis, plants exert considerable selective pressure on their fungal symbionts and are major drivers of diversification. This hypothesis was evaluated here by documenting the history of *Alnus*-ECM fungi association and specificity through molecular phylogenetic reconstructions.

Strong host-specificity patterns in mutualistic as well as parasitic relationships suggest intuitively a narrow coevolutionary history between symbionts [7]. The degree of parallel speciation and host switching between alders and three ECM taxa of basidiomycetes are explored as well as patterns of biogeography, with aims to develop more specific hypotheses on the processes that contribute to the diversification of the fungal lineages. Our predictions were that (i) the association with subgenus *Alnus*, more "modern" than subgenus. *Alnobetula*, is a derived character (the speciation of the fungi is linked to the speciation of the host), or (ii) evolution of a fungal symbiont would lead to increased specialization (there is an ongoing process of speciation by adaptation to new hosts).

The fungal lineages, *Alnicola *sect. *Alnicola *[32], *Alpova*, and *Lactariu*s, were selected on the following criteria: 1) are present with all species of *Alnus *(excluding *Paxillus *and *Gyrodon *for this reason, never reported with subgen. *Alnobetula*); 2) have well-defined species concept (excluding *Cortinarius*, *Tomentella *and *Alnicola *sect. *Submelinoides *[32] for this reason); 3) contain enough species to obtain an informative phylogenetic tree (excluding *Russula *and *Amanita *for this reason). Sequences from one to four DNA regions were analyzed, including the nuclear rDNA ITS, parts of the nuclear genes *rpb2 *and *gpd*, and the V9 domain of the mitochondrial SSU-rDNA. Phylogenetic relationships among the European alders (five species and subspecies) were investigated using the ITS and chloroplast MatK sequences.

## Results

### Host phylogeny and datation

**Figure 1:**
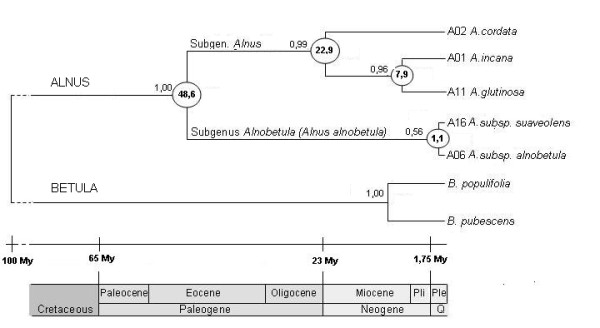
**Bayesian cladogram inferred from nuclear rDNA ITS and chloroplastic region MatK sequences of *Alnus *spp**. Numbers above branches are posterior probability scores. Numbers circled at the internodes are age in million years (My) estimated with Bayesian analysis implemented in BEAST software. The divergence between *Alnus *and *Betula *was assigned with an age of 95 to 105 My.

The phylogeny based on the combined data sets from ITS and MatK (Figure 1; see also Additional file 1: list of tree sequences used in this study) shows that European native species of *Alnus *are separated into two major clades that correspond to the subgenera *Alnobetula *and *Alnus*. Within the subgenus *Alnobetula*, *A. alnobetula *subsp. *alnobetula *and *A. alnobetula *subsp. *suaveolens *formed a monophyletic group. The subgenus *Alnus *clade comprises *A. glutinosa *and *A. incana. Alnus cordata *clusters outside this group. The multigene phylogeny confirms the relationship between the two *Alnus *clades with subgenus *Alnobetula *in a basal position. The molecular dating method gave the divergence between the two main subgenera of *Alnus *at the Eocene (around 48,6 My ago), and the original split between *A. cordata *and the *A. glutinosa*-*incana *complex at the Oligocene (around 22,9 My ago; Figure 1). Results also suggest recent lineage diversification at the Pleistocene for both subgenera (around 1,1 and 7,9 My ago for subgenus *Alnobetula *and the *A. glutinosa*-*incana *complex, respectively; Figure 1).

### *Alnicola *phylogeny and reconstruction of the history of associations

The ITS sequences for most *Alnicola *[abbreviated *Alc*. below] collections were complete (see Additional file 2: list of fungal sequences used in this study). However, the numbers of variable and informative characters of the ITS molecular dataset were very low (27 on ITS1 and 21 on ITS2 for the whole section, *i.e. *14 species). Most species were separated from each other by 2 to 6 nucleotides at best. Only *Alnicola pseudosalabertii *collections group together in a strongly supported clade that is well separated from the other lineages [32]. However, fourteen infrageneric groups (clusters or phylogroups, interpreted here as phylogenetic species) are identified, that are congruent with existing morphological taxonomy (morphospecies) at least for well-documented taxa [33,34]. Six of them could not be identified to valid taxa and are here cited under provisional names (*Alnicola badiofusca*, *Alc. citrinella*, *Alc. longicystis*, *Alc. pallidifolia*, *Alc. pseudosalabertii*, *Alc. xanthophylla*).

**Figure 2:**
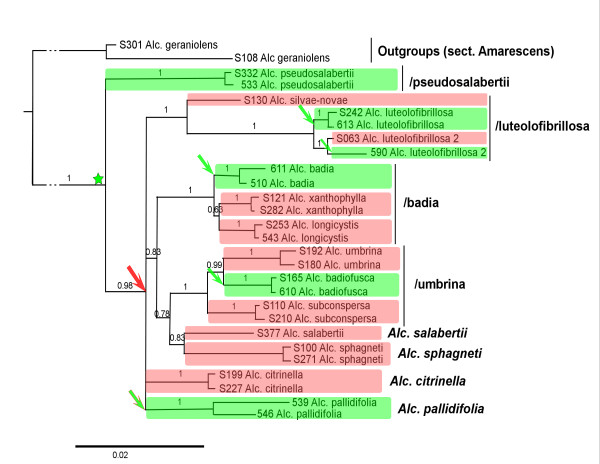
**Phylogenetic reconstruction of the *Alnicola *sect. *Alnicola *lineage (Hymenogastraceae) by concatenate analysis (ITS, GPD, RPB2, V9), showing putative evolutional events in relation with host specificity**. Red: species associated with *Alnus *sect. *Alnus*. Green: species associated with *Alnus *sect. *Alnobetula*. Green star shows putative basal association with *Alnus alnobetula *(possible co-speciation). Arrows show events of speciation associated with host specificity; red arrow: host-dependent radiation with *Alnus *subgen. *Alnus*; green arrow: host shifts from *Alnus *subgen. *Alnus *to *A. alnobetula*.

**Figure 3:**
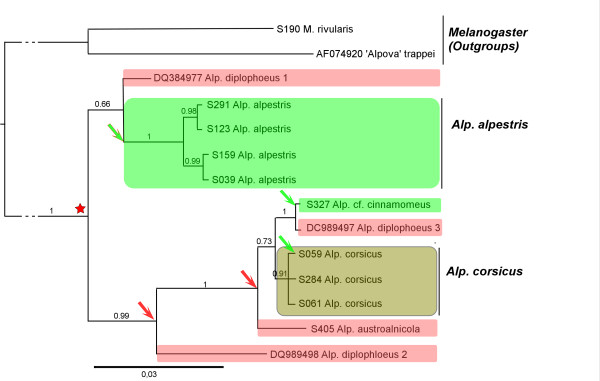
**Phylogenetic reconstruction of the *Alpova *lineage (Paxillaceae) based on ITS, showing putative evolutional events in relation with host specificity**. Red: species associated with *Alnus *sect. *Alnus*. Green: species associated with *Alnus *sect. *Alnobetula*. Brown: species associated with alders of both sections. Red star shows putative basal association with *Alnus sect. Alnus*. Arrows show events of speciation associated with host specificity; red arrow: host-dependent radiation with *Alnus *subgen. *Alnus*; green arrow: host shifts from *Alnus *subgen. *Alnus *to *A. alnobetula*.

**Figure 4:**
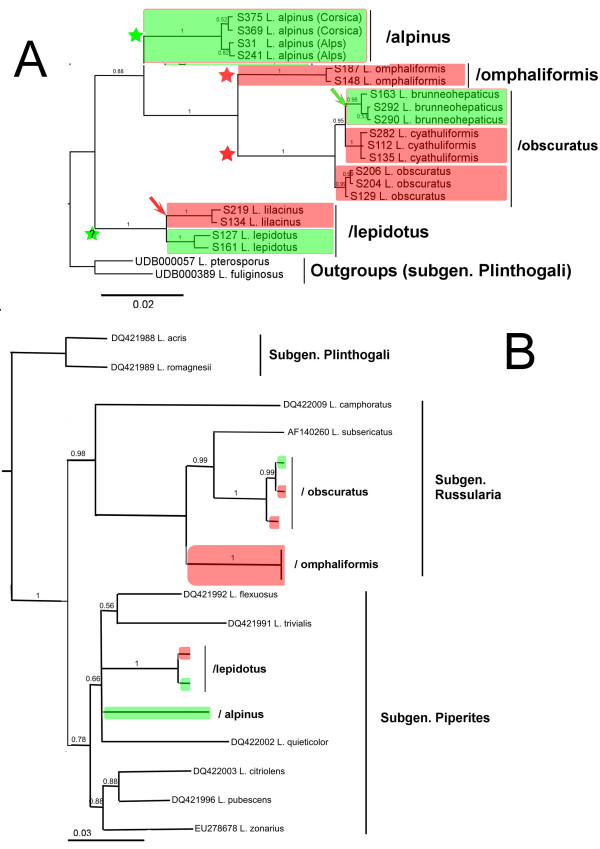
**Phylogenetic reconstructions of the *Lactarius *lineage (Russulaceae) based on ITS, showing putative evolutional events in relation with host specificity**. **A**: concatenate analysis of alnicolous species (ITS + RPB2 + V9). **B**: concatenate analysis (ITS + RPB2) of *Lactarius *subgen. *Russulares *showing positions of each clades of alnicolous species within the genus. Red: species associated with *Alnus *sect. *Alnus*.; Green: species associated with *Alnus *sect. *Alnobetula*. Green and red stars show putative basal association with *Alnus *sect. *Alnobetula *(with possible co-speciation) and sect. *Alnus *respectively. Arrows show events of speciation associated with host specificity; red arrow: host-dependent radiation with *Alnus *subgen. *Alnus*; green arrow: host shifts from *Alnus *subgen. *Alnus *to *A. alnobetula*.

Because the datasets of the four genes were highly congruent, a combined analysis was conducted on a 2743 bases long alignment, obtained by concatenation of all genes for each sampled morphospecies, with a Bayesian approach (Figure 2, 3, 4). *Alnicola geraniolens *Courtec., which belongs to *Alnicola *sect. *Amarescens *[33] (not associated with *Alnus*) was taken as an outgroup [32]. *Alnicola cholea *Kühner, in sect. *Cholea *P.A. Moreau [35], a more distant species not associated with *Alnus*, was also tested as outgroup but gave a weaker resolution on some branches (not shown). Although species relationships are still not completely resolved even when data from additional genes are included, the same fourteen species are clearly identified in all the analyses (each gene separately or the concatenated dataset), likely representing independently evolved lineages (Figure 2). The low sequence divergence and lack of phylogenetic resolution including with the combined gene dataset, suggests a fast radiation of these species in a short geological time.

The/pseudosalabertii clade is coming out as basal lineage amongst sect. *Alnicola*, with strong support in the concatenated analysis (Posterior Probability = 1, Figure 2). It is only represented by a rare species, only known from a few sites under *Alnus alnobetula *in mineral-rich subalpine situations. It has distinctive morphological characters such as spore features and pileal microstructure (unpublished data) that are also present in the non-alnicolous species of *Alnicola *sect. *Amarescens*, but not in other species of sect. *Alnicola*. These ancestral characters are congruent with its basal position in the phylogeny of *Alnicola *section *Alnicola. Alc. pseudosalabertii*, a species strictly associated to subgenus *Alnobetula*, appears clearly as an ancestral lineage.

The/luteolofibrillosa lineage appears as a collective species (or as a species complex), represented in Europe by at least two genetically distinct lineages. The former, *Alnicola luteolofibrillosa s. str*., is strictly associated with *Alnus alnobetula *in the Alps. The later, here named *Alc. luteolofibrillosa *"2", is collected under *Alnus *sect. *Alnus *in Europe and North America. Suprisingly, *Alc. luteolofibrillosa *"2" is found also with *Alnus alnobetula *subsp. *suaveolens *in Corsica, where *Alc. luteolofibrillosa s. str *would be expected according to host specificity. *Alnicola silvae-novae *(D.A. Reid) Courtec., a rare species encountered with *Alnus glutinosa*, comes out in the same cluster as *Alc. luteolofibrillosa *(Figure 2).

The ancestral state in the/luteolofibrillosa lineage is subgenus *Alnus*, while the host switches to *Alnobetula *probably occurred via a generalist species and niche contraction or host-dependent speciation (duplication).

In the/badia lineage, three species, *Alnicola badia *Kühner with *Alnus alnobetula*, *Alc. longicystis ad int. *and *Alc. xanthophylla ad int*. with *Alnus glutinosa *and *A. incana*, group together with very little nucleotide variation (7-8 characteristic positions on ITS1-2). All three are apparently restricted to specific soil conditions, *e.g. *sands and alluvial substrates.

The/umbrina lineage contains *Alnicola subconspersa *(P.D. Orton) Bon, *Alc. umbrina *(Maire) Kühner, and *Alc. badiofusca ad int*., that are three abundant fruiting species in alder stands. Both *Alc. subconspersa *and *Alc. umbrina *are associated with *Alnus glutinosa *and occasionally with *A. cordata *and *A. incana*, while *Alc. badiofusca *is found under *Alnus alnobetula*. In the present study, *Alnicola dubis *P.-A. Moreau & Vidonne is assimilated to *Alc. subconspersa*. Both *Alc. striatula *(P.D. Orton) Romagn. and *Alc. scolecina *(Fr.) Romagn. *s. auct. *[36] are assimilated to *Alc. umbrina. *Despite a recognition of *Alc. dubis*, *Alc. striatula *and *Alc. scolecina *in traditional taxonomy [34], these three species are temporarily grouped with the previous ones because of weak morphological support and only 1-2 nucleotide differences in the ITS sequences. These provisional taxonomic choices do not influence the results of our analysis since these taxa share the same host (*Alnus glutinosa*). However, taxonomy in this lineage obviously remains to be precised.

Finally four distinct species that comprise *Alnicola citrinella *(*Alc. escharioides *(Fr.: Fr.) Romagn. *s. auct. pl*.) with *Alnus glutinosa *and *A. incana*, *Alc. pallidifolia *with *A. alnobetula*, *Alc. salabertii *P.-A. Moreau & Guy Garcia with *A. cordata *[37], and *Alc. sphagneti *(P.D. Orton) Romagn., are also difficult to position in the phylogeny. However, they are characterized by distinctive morphological and ecological characters as well as nucleotide differences in the ITS and the other gene regions. *Alc. sphagneti *is associated with *Alnus glutinosa*, and apparently restricted to North Atlantic acidic localities; in the phylogenetic analyses, it comes out at the basis of the/umbrina lineage (Figure 2).

### *Alpova *phylogeny

The monophyletism of both genera *Alpova *[abbreviated *Alp. *below] and *Melanogaster *[28] is strongly supported by both ITS (PP = 1) and multigene phylogenies (Figure 3), and these results are consistent with previous studies [38,39]. *Melanogaster *is here taken as outgroup [40] to root the *Alpova *phylogeny. The relationships among the *Alpova *species were also well resolved with both phylogenies (not shown). Because *Alpova *appears as a genus with a reduced diversity in Europe (three species, Figure 3) the analysis was extended to extra-European species of *Alpova*, and phylogenetic reconstructions performed using ITS sequences.

Phylogenetic analysis of the ITS dataset clearly splits *Alpova *into two strongly supported clades, both including North American and European species (Figure 3). The first clade (PP = 0,83) contains an unidentified American taxon, *Alpova "diplophloeus *1", in a basal position, while *Alp. alpestris*, a European species often encountered under *Alnus alnobetula *in the Alps, Carpathians and Corsica, is placed in a derived position. No significant molecular difference was found among continental collections of *Alp. alpestris *(represented by 9 sporocarps from 4 localities, all located in the French Alps). The two Corsican collections (3 sporocarps from 2 localities) are separated from the mainland collections by 9 nucleotide positions: two in the ITS1, one in RPB2 and 6 in GPD. The second clade (PP = 1) is rooted by another unidentified American taxon (*Alp. "diplophloeus *2") and the South American *Alp. austroalnicola *associated with *Alnus acuminata *s.lat. (subgenus *Alnus*). It comprises three derived taxa that are the Corsican endemic *Alp. corsicus *(non host-specific but mainly with subgenus *Alnus*), *Alp. "diplophloeus *3" (DQ989497) under *Alnus incana*, and a European collection (*Alp. *cf. *cinnamomeus*) that has been found so far under *A. alnobetula*.

The case of *Alpova corsicus *deserves a particular attention as this species, represented here by 8 sporocarp collections from 6 sites in Corsica (see Additional file 2: list of fungal sequences used in this study) and one sequence from mycorrhiza (not shown), appears restricted to the island of Corsica. When found, it fruits abundantly under *Alnus glutinosa *in riparian and peaty forests, but also under *A. cordata *at supramediterranean level, and more occasionally under *A. alnobetula *subsp. *suaveolens *(one single collection with longer spores) [28]. Remarkably, our numerous prospects under *A. glutinosa *or *A. incana *in continental France never showed us any basidiome of *Alpova*. In addition to the information provided by sporocarp surveys, molecular studies of ECM communities have not revealed so far the presence of *Alpova *species in alder stands of *A. glutinosa *or *A. incana *elsewhere in continental Europe. The few reports of *Alpova *sporocarps from *Alnus incana *and *A. glutinosa *in central Europe are referred to *Melanogaster luteus *[28,41].

### *Lactarius *phylogeny

Collections of *Alnus*-associated *Lactarius *are grouped in four independent lineages, based on the combined molecular phylogeny of ITS and rpb2 sequences (Figure 4A, 4B) as well as on separated gene phylogenies (not shown). The internal systematics of *Lactarius *is still insufficiently documented and the nomenclature of subgenera and sections follows the most recent phylogenetic assessment of the whole genus [42,43]; *Alnus*-associated species belong to subgen. *Piperites *and subgen. *Russulares *which form together a monophyletic lineage. Subgenus *Plinthogali *is taken as outgroup for rooting the *Piperites*-*Russulares *phylogeny [42].

The/lepidotus lineage contains two species that are *Lactarius lepidotus *Hesler & A.H. Sm. strictly associated with *Alnus alnobetula*, and *L. lilacinus *(Lasch: Fr.) Fr. associated with *A. glutinosa *and *A. incana*. The two species are closely related despite strong morphological differences. Both are usually classified by taxonomists in subsection *Coloratini *(including species such as *L. helvus*, *L. glyciosmus*, and *L. alpinus*) due to the presence of typical dry squamulose pileus [44], but here surprisingly, they show genetic affinities with several groups with gelatinized pileus (ixocutis) of subgen. *Piperites*, represented in the phylogeny by sect. *Deliciosi *(*L. quieticolor*) (Figure 4B).

All collections of *L. alpinus *Peck, from the Alps and Corsica, group together with other species of *Lactarius *from subgen. *Piperites*, sect. *Glutinosi*, surprinsingly without close relationship with *L. lepidotus *despite morphological affinities (Figure 4B).

*L. alpinus *is associated to *Alnus alnobetula *and has been reported worldwide in the distribution range of its host, *e.g. *in N.E. USA, Alaska, Greenland, Carpathian Mountains [44,45]. *L. alpinus *var. *mitis *Hesler & A.H. Sm. is known from alder trees (subgen. *Alnus*) in Western U.S.A. [45], and might represent a possible host-shifted vicariant of *L. alpinus*; unfortunately no DNA sequence or reliable dry material could be obtained.

The two remaining lineages (/omphaliformis and/obscuratus) are more modern than the previous ones, coming out from a species-rich group of small *Lactarius *(subgen. *Russularia*) mostly common in temperate-subarctic areas, such as *L. tabidus *(Fr.: Fr.) Fr., *L. subsericatus *Bon, or *L. aurantiacus *Fr., and strongly host-specific such as *L. subdulcis *(Fr.: Fr.) Fr. (restricted to *Fagus*) and *L. hepaticus *Plowr. (restricted to *Pinus*).

*L. omphaliformis *Romagn. forms a single clade that is genetically very stable, and surprisingly comprises a sequence obtained from *Alnus acuminata *in Argentina (GenBank DQ195543). In Europe, *L. omphaliformis *has been reported from the West under *A. glutinosa *in typically acidic situations, but it might be a cosmopolitan species associated with more species of *Alnus *sect. *Alnus *worldwide. The lack of reports under *A. incana *could be due to narrow biogeographical and edaphic preferences, not compatible with the natural distribution of the host in Europe. It is also one of the very few symbiotic species of *Alnus *sect. *Alnus *for which no relative species is known on *A. alnobetula*.

The/obscuratus lineage is composed of morphologically highly variable and taxonomically confused taxa (Figure 4A). Six distinct species or varieties are recognized by mycologists [44,46,47] and many names are usually cited in literature (*e.g. L. obscuratus *Lasch: Fr., *L. cyathuliformis *Bon, *L. clethrophilus *Romagn., *L. brunneohepaticus *M.M. Moser, *L. obscuratus *var. *radiatus *(J.E. Lange) Romagn., *L. obscuratus *var. *subalpinus *Basso, *L. radiatus *var. *alnobetulae *Bon). In the present study, three well-separated phylogroups are identified with a clear host specificity (Figure 4A) Both *L. obscuratus s. str*. (including *L. clethrophilus*) and *L. cyathuliformis *are found in association with *A. glutinosa*, *A. incana *and *A. cordata*, while *L. brunneohepaticus *(including *L. obscuratus *var. *subalpinus*) is associated with *A. alnobetula *in the Alps and Corsica.

## Discussion

### Looking for early speciation events

The ancestral position of subgenus *Alnobetula *inferred from worldwide molecular phylogenies of alders [11,12] is confirmed by our results. Our molecular clock estimates are consistent with the fossil records that suggest an extant ancestor of *A. cordata*-*glutinosa*-*incana *at the Oligocene (around 22,9 Mya) and recent radiation events at the Pleistocene in the subgenus *Alnobetula *and the *glutinosa*-*incana *complex (Figure 1). A low sequence divergence was also cited [11] in the ITS region within the subgenus *Alnobetula *and the *A. incana *species complex (including *A. glutinosa*), indicating recent diversification in the circumpolar areas.

*Alnicola *is the most informative genus thanks to its species richness. Since no supraspecific classification in sect. *Alnicola *has been proposed so far in taxonomic literature, the present phylogeny will contribute to a better systematic and taxonomic treatment of the genus (unpublished results). *Alnicola pseudosalabertii*, a species with ancestral morphological characters, is likely the oldest known *Alnus*-associated species in the *Hymenogastraceae *family. Its basal position in the *Alnicola *phylogeny suggests a common origin of the *Alnus*-associated fungal lineage and the most ancestral *Alnus *lineage, and therefore a synchrony in the occurrence of both lineages. However, the absence of fossils or standardized molecular clock in fungi did not allow us to test the time concordance between the plant and the fungal lineages.

In *Lactarius*, the two oldest *Alnus*-associated lineages root at the basal origin of temperate *Lactarii *(Figure 4B) and are likely to find their origin at the same early period of emergence of temperate-subarctic *Alnus *species of subgen. *Alnobetula *[48]. This is congruent with the fact that *Lactarius lepidotus *(/lepidotus) and *L. alpinus *(/alpinus), two widespread species with ancestral morphological characters in the genus (dry trichodermial pileus structure) are strictly associated with *Alnus alnobetula *worldwide. *L. lilacinus*, here appearing as a sister clade of *L. lepidotus *(Figure 4A), would be an example of phylogenetic speciation with *Alnus *sect. *Alnus *if it could be shown to derive from *L. lepidotus*.

In *Alpova*, the two species currently known to be associated with *Alnus alnobetula *(*Alp. alpestris *and *Alp*. aff. *cinnamomeus*) are in derived position in the phylogeny (Figure 3), and therefore do not illustrate any co-evolutive pattern (but information is lacking about host identity of North American species). Hypothesis of a recent emergence of the *Alpova *lineage with *Alnus *sect. *Alnus *cannot be excluded. The alternative hypothesis is an older origin of *Alpova *with *Alnus alnobetula *with host-shift and later radiation with *Alnus *sect. *Alnus*. If so, then ancestral species that would be analogous to *Alnicola pseudosalabertii *in the *Alnicola *phylogeny, should be found under *Alnus alnobetula *in and outside Europe.

### Recent host-dependent evolution processes

If the hypothesis of co-speciation could explain the basal topology of the oldest *Alnus*-associated lineages such as/pseudosalabertii (*Alnicola*),/lepidotus (*Lactarius*) and/alpinus (*Lactarius*), the prediction of a specific diversity only driven by co-evolution processes in which ancestral species would be associated to *Alnus alnobetula*, and recent lineages to *Alnus glutinosa*-*A. incana*, is clearly not congruent with the current species diversity observed in *Alnus*-associated communities. Except *Lactarius *species (*L. alpinus *and *L. lepidotus*) which are still dominant in *Alnus alnobetula *ecosystems, most ECM fungal species currently dominating the community (such as *Alpova alpestris*, *Lactarius brunneohepaticus*, *Alnicola badia*, *Alc. badiofusca*, *Alc. luteolofibrillosa*) are derived from fungal lineages evolved with *Alnus *sect. *Alnus *(Figure 1). In *Alnicola*, the main current diversity likely appears during a fast radiation period. It is tempting to compare this diversification event to the fast radiation period of *Alnus *during the Pleistocene [11,12], which suggests that recent lineages of fungi show the same evolutionary patterns that *Alnus *sect. *Alnus*. Similarly to *Betula*-associated *Leccinum *species (*Boletales*) [6], the most plausible mechanism for this diversification event is allopatric speciation with isolation of fungal populations in glacial refuges of their host at the Pleistocene.

### Host shifts but not host extension?

The two hypotheses previously suggested - early co-evolution with *Alnus *sect. *Alnobetula*, and later host-dependent speciation with *Alnus *sect. *Alnus*, explain correctly most of the topology of the three fungal genera, but the presence in derived branches of species associated with *Alnus alnobetula *- not explained by any co-evolution scheme - suggests another factor of host-dependent speciation. Most species associated with *Alnus alnobetula *derived from lineages associated to *Alnus *sect. *Alnus *(*e.g. Alnicola badia *in the/badia lineage, and *Alc. badiofusca *in the/umbrina lineage; *Lactarius brunneohepaticus *in the/obscuratus lineage; and *Alpova alpestris *derived from *Alnus *sect. *Alnus *associated species). The only explanation for so many examples of recent *Alnobetula*-associated species of ECM fungi is recent host-shifts that have occurred independently in most fungal lineages, from modern species of *Alnus *subgen. *Alnus *to *A. alnobetula*, followed by a fast host-dependent speciation. Interestingly enough, no example has been found of host shift from *Alnus alnobetula to A. glutinosa*/*A. incana *hosts. This unilateral phenomenon suggests that environmental pressure or promiscuity of different species of *Alnus *cannot explain completely such host-shift phenomena, which certainly implies important genetic mechanisms of host recognition so far unidentified.

### What Corsica tells us about host shifts

The geographic co-expansion of *Alnus alnobetula *and *A. incana *throughout their distribution area since the Pleistocene [11] and their frequent co-occurrence in the same geographical areas (*e.g. *in the Alps where *A. alnobetula *and *A. incana *co-exist and grow locally intermixed) create environmental contexts that should facilitate host-shifts. If colonization of new hosts is more a matter of geographic proximity than relatedness of the hosts, then numerous derived species and unsolved species complexes would be expected. The results show that recent jumps to distantly related hosts (from different subgenera) are relatively infrequent and limited to one "shifted" new species per lineage (see above). Fungal species with broad host range are not observed in derived positions of the three fungal phylogenies. All studied species are well resolved at specific level and highly host-specific at the level of plant subgenus (even when collected in sites where alders from the two subgenera grow adjacent to each other, as shown by molecular analysis of mycorrhizae, Rochet *et al. *unpublished data). This pattern of host-symbiont association is also observed in '*Alnicola*' sect. *Submelinoides *[32 and unpublished data], *Russula *(J. Borovička, unpublished data), *Cortinarius *[30], and several other genera [49]. By contrast, colonization of close relatives within each subgenus of *Alnus *is occurring frequently, possibly explained by host tracking through hybrids. Thus, the same species of ECM fungi are commonly found under *A. glutinosa *and *A. incana*, two alder species for which natural hybridization has been reported in the boundaries of their distribution areas [50]. Such a scenario of host-tracking coevolution (that could eventually lead to speciation) have been nicely shown between *Puccinia *rust fungi and their crucifer (*Brassicaceae*) hosts [51].

Corsica is a remarkable exception to this general pattern of host-symbiont association. Thus, host jumping to distantly related hosts appears to be facilitated in Corsica because there are at least three species of fungi that are commonly found under *A. glutinosa *(*A. incana *never existed in Corsica) and *A. alnobetula *subsp. *suaveolens*, despite the geographical distance between collections from the two hosts (at least 10 km apart from each other). The generalist species are *Alnicola luteolofibrillosa "2" *(with an already perceptible genetic divergence of 2 nucleotide positions on ITS1 between *A. glutinosa*- and *A. alnobetula *subsp. *suaveolens*-associated populations), *Alpova corsicus *[28], and *"Alnicola" inculta *(Peck) Singer (sect. *Submelinoides*, not treated in this study) present in Corsica under *A. alnobetula *but never found or reported from the Alps under this tree so far.

It is likely that the speciation process by host shift which led to the differentiation of most *Alnus alnobetula*-associated fungi from *Alnus incana*-*glutinosa *-associated lineages, is still active, or at last much more recent or less advanced, in Corsica where the three examples cited above seem to illustrate a case of "host extension" preceding speciation. In contrast to the "*Leccinum *model" [6], "host extension" here does not extend to unrelated new hosts but to another species of *Alnus*, and is probably favoured by tree promiscuity as well as by insularity and genetic isolation from the continent. More examples would have to be looked for in other areas of Quaternarian glacial refugia where *A. glutinosa*/*A. incana *and *A. alnobetula *still co-exist, *e.g. *in Eastern Europe or Caucasus. As real islands provide an opportunity to examine the factors causing evolution in ECM fungi [52], fine-scale genetic structure of the fungal species present on the mainland, Corsica, and other host Mediterranean islands (*e.g. *Sardinia) should also be pursued in future studies.

### The *Alnus*-ECM fungal symbiosis: a powerful model for the study of speciation of ECM fungi

The factors driving ECM diversity in terms of speciation processes remain poorly documented, and only a few examples have been satisfactorily investigated. The interactions between alders and ectomycorrhizal fungi appear highly specialized, and as a consequence when hosts undergo certain selection regimes, their symbionts might also take evolutionary steps to maximize their fitness. The evolutionary framework developed in this study gives an alternative to the cyclic-biphasic model developed on *Leccinum *(ECM Boletales) [6], which implies more or less soft periods of host contractions and extensions driven by environmental changes, in which coevolution does not act significantly.

Because coevolutionary events become masked through time via host switching, extinction and duplication, finding empirical evidence for co-speciation (or co-divergence) patterns has proven difficult in many groups of organisms, including classical examples of animal parasites. However, possibly due to the very selective pressure of the host, ECM coevolution appears in *Alnicola *and in *Lactarius *as a basal factor of speciation in these genera in relation with the subgeneric diversification of *Alnus*. More investigations on extra-European *Alnus*-associated communities are necessary to fill the phylogenetic puzzle in all these genera and confirm the reality of this early speciation process. Evidence for ongoing local coevolutionary selection could also be obtained by experimental pairings between plant and fungal populations [53].

The second element of the historical associations between plants and fungal symbionts is a host-dependent speciation (radiation without host change), suggested marginally [6] for explaining the radiation of a *Betula*-associated group of *Leccinum*, here observed clearly in *Alnicola *and *Alpova *in relation with *Alnus *sect. *Alnus*; allopatry is a possible cause of genetic isolation of populations evolving quickly as genetically distinct species (detectable by unsolved rake-like clades). This phenomenon of Pleistocene origin might have driven active speciation processes in relation with *Alnus *sect. *Alnus*. A fine-scale genetic study of common fungal species associated e.g. with *Alnus glutinosa *[15] in its glaciation refugia such as Spain, North Africa, Corsica, Balkans and Turkey, would help quantifying local host-dependent diversification processes in fungi, migration and interbreeding processes at a continental scale, and extrapolate them to more ancient speciation events.

Host shifts from *Alnus alnobetula *to subgen. *Alnus *are observed in most lineages of *Alnicola *(at least four times), *Alpova *(twice) and *Lactarius *(once) (Figure 2, 3, 4), but they do not represent such a common event as could be expected by current geographic proximity of trees from the two subgenera, or by their assumed promiscuity in Pleistocene refuges. Other, probably rare factors are necessarily involved to explain this phenomenon. Host shift is likely a consequence of past host extension; in our study we have identified active or very recent host extensions in Corsica, of fungi usually associated with *Alnus glutinosa *locally extending to *A. alnobetula *subsp. *suaveolens *without significant genetic or morphological deviation. In Corsica the current coexistence of these trees at short distance, and even their close promiscuity during glaciations events, are reinforced by insularity and confinement in narrow valleys. In the Alps this phenomenon might be older than in Corsica, since sister species associated with *A. alnobetula *are genetically and morphologically well differentiated from those associated to the *A. glutinosa*-*A. incana *complex.

## Conclusions

Although extreme specialization may represent an evolutionary-dead-end, host-dependent speciation seems to have played a more important role than host shift across large phylogenetic distances in the evolution of the studied fungi, as especially illustrated by *Alnicola*, the most species-rich studied group. The *Alnus*-EcM fungi communities are a unique model for reconstituting the events of speciation amongst symbiotic fungi. Fungal assemblages associated with alders comprise other genera such as *Tomentella *(Thelephorales), *Cortinarius *(Agaricales), *Paxillus *(Boletales), *Russula *(Russulales), which will complete the present study.

Inferences about the evolutionary events of symbionts in the light of phylogenies are sensitive to the information at hand. By adding missing or newly discovered fungal species to the trees, especially from the numerous non-investigated alder species outside Europe, our picture about the details of coevolution or host switching may change. Advancing the work presented here would benefit from additional sampling of the fungi (sporocarps and mycorrhizae), particularly in areas that would introduce new host associations not represented here. Although estimation of the time of divergence of the clades will always be difficult in fungi due to the lack of fossils, what is also required is a comprehensive and well calibrated tree based on numerous molecular divergence rate estimates. Most such data are not available today. A concerted effort is thus necessary for understanding the evolution of *Alnus-*associated fungi, what in turn would greatly benefit to the understanding of the effects of evolutionary and historical events in the late Tertiary and Quaternary on the geographic distribution of ECM fungi in general [54], with transferability of this information to biodiversity studies and conservation programs.

The present study focused on historical events and co-evolutionary relationships in explaining current patterns of host specificity in the *Alnus *ECM symbiosis. Further research is needed to shed light on the role of ecological parameters such as soil nitrogen conditions in shaping the distinct nature of *Alnus *ectomycorrhizal assemblages and the current distribution of the fungal species.

## Methods

### Taxonomic sampling

#### PLANTS

The four European native species of alders (= five species and subspecies) are listed in Additional file 1, along with the gene regions analyzed for each of them and GenBank accession numbers.

#### BASIDIOMYCOTA

**Figure 5:**
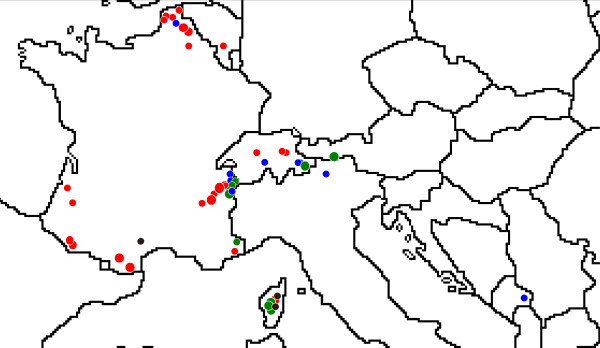
**Mapping of sampled sites for sporocarps in Western and Central Europe (for details see Additional file **2**)**. Colour of circles indicates tree species; green: *Alnus alnobetula*; black: *A. cordata*; red = *A. glutinosa*; blue: *A. incana*. Large circles: at least three species sampled (sites visited for this study); small circles: one or two species sampled (occasional visits on sites or collections sent by correspondents).

Most fungal sequences used in this study are derived from extensive sporocarps sampling of *Alnicola*, *Alpova *and *Lactarius *conducted by PAM over several years. Sporocarps of 220 *Alnicola*, 61 *Lactarius *and 29 *Alpova *were collected from multiple stands of the four European alder species, at various locations in France and other European countries (especially Austria and Switzerland; Figure 5). In most cases, host could be designated unambiguously in the field, one species of alder trees being locally the only ECM host plants, but in a few cases the closest ECM host tree was design as the host plant. Molecular typing of ectomycorrhizae was also performed to confirm the identity of the host and the fungal symbiont (the results will be presented elsewhere). Tissue samples (gills or part of the fruitbody flesh) were stored in a CTAB 2X Buffer (100 mM Tris-HCl (pH 8), 1.4 M NaCl, 20 mM Na2 EDTA, 2% CTAB) until DNA extraction. Taxonomy and nomenclature follow [28] for *Alpova*, [42] for *Lactarius*, [34] and partly unpublished results for *Alnicola*. Voucher specimens are deposited at the Herbarium of the Faculté des Sciences Pharmaceutiques et Biologiques, Lille (LIP), France. The dataset also included sequences from earlier studies [32,55,42,58] and some unpublished sequences with reference material downloaded from public databases (GenBank, UNITE). Information regarding the sampling (species, host plant, geographic location, DNA and herbarium collection numbers, genes analyzed, GenBank accession numbers, references) is provided for all materials in Additional file 2: list of fungal sequences used in this study as supplementary data.

### DNA extraction, PCR amplification and sequencing

Total DNA was extracted from *Alnus *leaves, CTAB samples and herbarium specimens of ECM fungi using the kit *Wizard Genomic DNA Purification Kit *(Promega) according to the manufacturer's protocol.

#### PLANTS

Two DNA regions were selected for the present *Alnus *phylogeny: the nuclear rDNA ITS and the chloroplastic region MatK. The PCR reaction mix included 0.2 μl of Go*Taq *5 U/μl (Promega), 10 μl of 5X buffer, 1 μl of 20 μM for each primer, 1 μl of dNTP 10 mM, 1 μl of DNA template and nuclease free water for a final volume of 50 μl. Standard cycling parameters were an initial denaturation step at 94°C for 1 minute, 40 cycles of denaturation at 94°C for 30 s, annealing for 40 s at different temperatures depending on the primer set (57°C for ITS, 46°C for matK) and elongation at 72°C for 40 s, followed by a final elongation step at 72°C for 5 min.

#### BASIDIOMYCOTA

One to four regions were amplified and sequenced depending on the genus and sample. The DNA regions and primers used for PCR are the nuclear rDNA ITS using the primer set ITS-1F/ITS-4B [59], the rpb2 gene using bRPB2-5F/bRPB2-7.1R [60], the gpd gene using GPD-F/GPD-RA [61,62], and the hyper variable domain V9 of the mitochondrial SSU-rDNA using the V9U/V9R [63,64].

Amplifications were carried out in 25 μl reaction containing 0,2 μl of Go*Taq *polymerase of 5 U/μl (Promega), 5 μl of Buffer 5X (Promega), 0.25 μl of 5 μM for each primer, 5 μl of dNTPs 5 mM, 2 μl of template DNA and 15.8 μl of nuclease free water. Standard cycling parameters were an initial denaturation step at 94°C for 3 min, 35 cycles of denaturation at 94°C for 45 s, annealing for 45 s at different temperatures depending on the primer set (50° for rpb2, 53° for V9, and 55°C for ITS and gpd), and elongation at 72°C for 1 min, followed by a final elongation step at 72°C for 10 min. Post-cycling, samples were held at 4°C.

PCR products were loaded onto a 1.25% standard agarose gel for electrophoresis (20 min at 100 W). Gels were stained with ethidium bromide and photographed under ultraviolet light. Unsuccessfully amplified samples were subjected to multiple amplifications at various DNA concentrations. Sequencing was done by MilleGen (Labège, France).

### Alignment and phylogenetic analyses

Sequences were manually edited using Sequencher 4.8. Gene Codes (Ann Arbor, MI). They were aligned using MAFFT version6 using the LINS-i method with standard settings [65] and subsequently carefully refined by eye. Phylogenetic analyses were conducted on sequence datasets from individual and combined DNA regions using maximum likelihood reconstruction with PhyML [66] and Bayesian inference as implemented in MrBayes 3.1.2 [67].

#### MAXIMUM LIKELIHOOD

In PhyML, trees were constructed for each DNA region using the most general time-reversible model of nucleotide evolution with Gamma distributed errors on mutation rates (GTR+G) and node support was estimated by using the approximate likelihood-ratio test (alrt), a much faster method for estimating branch support than either the bootstrap or Bayesian posterior probabilities [51].

#### BAYESIAN INFERENCE

Modeltest 3.7 [68] was run for all data sets (individual and combined) to select a model of sequence evolution. Appropriate DNA substitution models and its parameters were determined based on the comparisons of negative log likelihood values in Modeltest 3.7 [69] implemented in PAUP*4.0b10 [70]. Likelihood and prior settings were changed to meet with the settings corresponding to the models found for each marker. Gaps were coded as missing data. The analyses were initiated with random starting trees and one cold and three incrementally heated Markov chain Monte Carlo (MCMC) chains were run for 10,000,000 generations. Trees were sampled every 1000 generations. The previously determined model of sequence evolution to each data set was applied in the partitioned Bayesian analyses for all combined data sets. For each data set, MCMC runs were repeated twice. Stationarity of the Markov chain was ascertained by plotting likelihood values against number of generations for apparent stationarity. The first 1000 to 2000 trees were discarded as burn-in, and the remaining trees were used to calculate a 50% majority rule tree and to determine the posterior probabilities for the individual branches.

### Estimation of time divergence in *Alnus *phylogeny

To estimate divergence times between lineages, the combined data set of ITS and MatK sequences was employed. Rate constancy was tested with a likelihood ratio test [71]. Because the molecular data departed from clock-like evolution, a Bayesian analysis implemented with BEAST ver. 1.4.7 [72] was used under a log-normal relaxed molecular clock [73] and a Yule pure birth model of speciation to estimate the times of divergence and their credibility intervals in the genus *Alnus*. Posterior distributions of parameters were approximated using two independent MCMC analyses of 10,000,000 generations with 10% burn-in. Samples from the two runs which yielded similar results were combined and convergence of the chains was checked using the program Tracer 1.3 [74]. Based on a recent *Betulaceae *phylogeny [75] the divergence between *Alnus *and *Betula *was assigned with an age of 95 to 105 million year (My).

## Abbreviations

ad int.: *ad interim *(provisional names for unpublished taxa); *Alc.*: *Alnicola*; *Alp.*: *Alpova*; ECM: ectomycorrhizal (fungi forming ectomycorrhizae with Tracheophytes).; My: 10^6 ^years; s.lat.: *sensu lato*; s.str.: *sensu strict*; sect.: section (taxonomic rank); subgen.: subgenus (taxonomic rank)

## Authors' contributions

JR carried out the molecular phylogenies, and participated in writing of the manuscript as a part of her PhD project. PAM carried out most of the sporocarp samplings and taxonomic identifications, and was deeply involved in the writing of the manuscript along with MG. SM did most of the DNA extractions from sporocarps, the PCRs and the editing of the sequences. MG's contributions are the conception and the coordination of the study, the acquisition and the analysis of the data, and the writing of the manuscript. All authors read and approved the final manuscript.

## Supplementary Material

Additional file 1 **List of tree sequences used in this study**. Sequences of *Alnus *and *Betula *species are either generated by the authors or downloaded from GenBank. Vouchers (leaves) are preserved at the Laboratoire Evolution et Diversité Biologique, Toulouse (F). Click here for file

Additional file 2 **List of fungal sequences used in this study**. Sequences are either generated by the authors (402 sequences) or downloaded from GenBank or UNITE databases (89 sequences). ^**(1)**^: not a genuine *Alpova *but a *Melanogaster *[28]. Abbreviations of host names: **Aacum**: *Alnus acuminata*; **Aalnobet**: *Alnus alnobetula*; **Acord**: *Alnus cordata*; **Afrut**: *Alnus fruticulosa*; **Aglut**: *Alnus glutinosa*; Ainc: *Alnus incana*; **Arub**: *Alnus rubra*; **Arub?**: likely *Alnus rubra *(M. Berbee, personal communication); **Asuav**: *Alnus alnobetula *subsp. *suaveolens*; **Asp**.: unidentified species of *Alnus*. Abbreviations of countries: **A**: Austria; **ARG**: Argentina; **CAN**: Canada; **CH**: Switzerland; **CZ**: Czech republic; **F**: France; **GB**: Great Britain; **HU**: Hungary; **I**: Italy; **MTN**: Montenegro; **RUS**: Russia; **N**: Norway; **SW**: Sweden; **USA**: United States of America. Click here for file

## References

[B1] Smith SE Read DJ Mycorrhizal Symbiosis 2008 3 London: Academic Press

[B2] Molina R Massicotte H Trappe JM Allen MF Specificity phenomena in mycorrhizal symbioses: community-ecological consequences and practical implications Mycorrhizal symbiosis, an integrative plant-fungal process 1992 New York: Chapman and Hall 357 423

[B3] Roy M Dubois MP Proffit M Vincenot L Desmaris E Selosse MA Evidence from population genetics that the ectomycorrhizal basidiomycete *Laccaria amethystina *is an actual multihost symbiont Mol Ecol 2008 17 2825 2838 10.1111/j.1365-294X.2008.03790.x 18489549

[B4] Kretzer A Li Y Szaro T Bruns TD Internal transcribed spacer sequences from 38 recognized species of *Suillus *sensu lato: phylogenetic and taxonomic implications Mycologia 1996 88 776 785 10.2307/3760972

[B5] Bruns TD Bidartondo M Taylor DL Host specificity in ectomycorrhizal communities: what do exceptions tell us? Integ Comp Biol 2002 42 352 359 10.1093/icb/42.2.352 21708728

[B6] Den Bakker HC Zuccarello GC Kuyper TW Noordeloos ME Evolution and host specificity in the ectomycorrhizal genus *Leccinum* New Phytol 2004 163 201 215 10.1111/j.1469-8137.2004.01090.x 33873790

[B7] Thompson JN The geographic mosaic of coevolution 2005 Chicago: University of Chicago Press

[B8] Furlow JJ The systematics of the American species of *Alnus *(Betulaceae) Rhodora 1979 81 1 121

[B9] Kvaček Z Teodoridis V Tertiary macrofloras of the Bohemian Massif: a review with correlations within Boreal and Central Europe Bull Geosci 2007 82 383 408

[B10] Schmidt PA Zur Systematik und Variabilität der mitteleuropäischen Erlen (Gattung *Alnus *Mill.) Mitt Deutsch Dendrol Ges 1996 82 15 42

[B11] Chen Z Li J Phylogenetics and biogeography of *Alnus *(Betulaceae) inferred from sequences of nuclear ribosomal DNA ITS region Int J Pl Sci 2004 165 325 335 10.1086/382795

[B12] Navarro E Bousquet J Moiroud A Munive A Piou D Normand P Molecular phylogeny of *Alnus *(Betulaceae), inferred from ribosomal DNA ITS sequences Pl Soil 2003 254 207 217 10.1023/A:1024978409187

[B13] Murai S Phytotaxonomical and geobotanical studies on genus *Alnus *in Japan (III). Taxonomy of whole world species and distribution of each section Bull Gov Forest Exp Sta Meguro 1964 171 1 107

[B14] Kovar-Eder J Kvaček Z Martinetto E Roiron P Late Miocene to Early Pliocene vegetation of southern Europe (7-4Ma) as reflected in the megafossil plant record Palaeogeogr Palaeoclimatol Palaeoecol 2006 238 321 339 10.1016/j.palaeo.2006.03.031

[B15] King RA Ferris C Chloroplast DNA phylogeography of *Alnus glutinosa *(L.) Gaertn Mol Ecol 1998 7 1151 1161 10.1046/j.1365-294x.1998.00432.x

[B16] Hewitt GM Post-glacial re-colonization of European biota Biol J Linn Soc 1999 68 87 112 10.1111/j.1095-8312.1999.tb01160.x

[B17] Briquet J Prodrome de la flore corse, comprenant les résultats botaniques de six voyages exécutés en Corse sous les auspices de M. Émile Burnat 1910 Tome I. Geneva, Basel: Georg & Cie

[B18] Ball PW Tutin TG, Heywood VH, Burges NA, Valentine DH, Moore DM *Alnus *Miller Flora Europaea Psilotaceae to Platanaceae 1993 I 2 Cambridge University Press 69 70

[B19] Kamruzzahan S Is *Alnus viridis *a glacial relict in the Black Forest ? Master thesis 2003 Albert-Ludwigs Univ. Freiburg http://www.freidok.uni-freiburg.de/volltexte/1189/pdf/Is_Alnus_viri.pdf

[B20] Contandriopoulos J La flore orophile de la Corse: origines, rapports avec celle des Alpes et des montagnes de l'Europe méridionale Actes du colloque sur la flore et la végétation des chaînes alpine et jurassienne. Annales littéraires de l'université de Besançon 1971 Besançon: Presses Universitaires de Franche-Comté 205 222

[B21] Schönswetter P Tribsch A Vicariance and dispersal in the alpine perennial *Bupleurum stellatum *L. (Apiaceae) Taxon 2005 54 725 732

[B22] Molina R Ectomycorrhizal specificity in the genus *Alnus* Can J Bot 1981 59 325 334

[B23] Brunner I Brunner F Laursen GA Characterization and comparison of macrofungal communities in an *Alnus tenuifolia *and an *Alnus crispa *forest in Alaska Can J Bot 1992 70 1247 1258 10.1139/b92-158

[B24] Miller SL Koo CD Molina R Characterization of red alder mycorrhizae: a preface to monitoring belowground ecological responses Can J Bot 1991 69 516 531 10.1139/b91-071

[B25] Becerra A Zak MR Horton TR Micolini J Ectomycorrhizal and arbuscular mycorrhizal colonization of *Alnus acuminata *from Calilegua National Park (Argentina) Mycologia 2005 15 525 531 10.1007/s00572-005-0360-7 16034621

[B26] Tedersoo L Suvi T Jairus T Ostonen I Põlme S Revisiting ectomycorrhizal fungi of the genus *Alnus*: differential host specificity, diversity and determinants of the fungal community New Phytol 2009 182 727 735 10.1111/j.1469-8137.2009.02792.x 19320837

[B27] Kennedy PG Hill LT A molecular and phylogenetic analysis of the structure and specificity of *Alnus rubra *ectomycorrhizal assemblages Fungal Ecol 2010 3 195 204 10.1016/j.funeco.2009.08.005

[B28] Moreau PA Rochet J Richard F Manzi S Chassagne F Gardes M *Alnus*-associated species of *Alpova *and *Melanogaster *(Boletales, Paxillaceae) in Europe Cryptog Mycol 2011 in press

[B29] Trappe J Fungus associates of ectotrophic mycorrhizae Bot Rev 1962 28 538 606 10.1007/BF02868758

[B30] Moser M Some aspects of *Cortinarius *associated with *Alnus* J JEC 2004 3 47 101

[B31] Mohatt KR Cripps CL Lavin M Ectomycorrhizal fungi of whitebark pine (a tree in peril) revealed by sporocarps and molecular analysis of mycorrhizae from treeline forests in the Greater Yellowstone Ecosystem Botany 2008 86 14 25 10.1139/B07-107

[B32] Moreau PA Peintner U Gardes M Phylogeny of the ectomycorrhizal mushroom genus *Alnicola *(Basidiomycota, Cortinariaceae) based on rDNA sequences with special emphasis on host specificity and morphological characters Mol Phylogen Evol 2006 38 794 807 10.1016/j.ympev.2005.10.008 16314113

[B33] Moreau PA A nomenclatural revision of the genus *Alnicola* Fungal Div 2005 20 121 155

[B34] Horak E Röhrlinge und Blätterpilze in Europa - unter der Mitarbeit von Anton Hausknecht (Bolbitiaceae) und P.A. Moreau (*Alnicola*) 2005 Heidelberg: Elsevier Spektrum Akademischer

[B35] Moreau PA Mleczko P Ronikier M Ronikier A Rediscovery of *Alnicola cholea *(Cortinariaceae): taxonomic revision and description of its mycorrhiza with *Polygonum viviparum* Mycologia 2006 98 468 478 10.3852/mycologia.98.3.468 17040076

[B36] Romagnesi H Description de quelques espèces d'Agarics ochrosporés Bull Trimestriel Soc Mycol France 1942 58 121 169

[B37] Moreau PA Garcia G *Alnicola salabertii*, sp. nov., mycorhizique d'*Alnus cordata*, et deux autres *Alnicola *à petites spores Bull Trimestriel Soc Mycol France 2005 120 273 292

[B38] Grubisha LC Bergemann SE Bruns TD Host islands within the California Northern Channel Islands create fine-scale genetic structure in two sympatric species of the symbiotic ectomycorrhizal fungus *Rhizopogon* Mol Ecol 2007 16 1811 1822 10.1111/j.1365-294X.2007.03264.x 17444894

[B39] Binder M Hibbett DS Molecular systematics and biological diversification of *Boletales* Mycologia 2006 98 971 981 10.3852/mycologia.98.6.971 17486973

[B40] Halász K Különböző stressztűrőképességű nagygombanemzetségek Kárpát-medencei leletanyagának molekuláris azonosítása és rendszerezése PhD Thesis 2008 University of Budapest http://teo.elte.hu/minosites/ertekezes2009/halasz_k.pdf

[B41] Perič B Moreau PA *Melanogaster luteus*, un hypogé rare retrouvé au Monténégro Mycol Montenegrina 2010 12 77 83

[B42] Buyck B Hofstetter V Eberhardt U Verbeken A Kauff F Walking the thin line between *Russula *and *Lactarius*: the dilemma of *Russula *subsect. *Ochricompactae* Fungal Div 28 15 40

[B43] Heilmann-Clausen J Verbeken A Vesterholt J The genus Lactarius. Fungi of Northern Europe 2 1998 Copenhagen: The Danish Mycological Society

[B44] Basso MT Lactarius Pers 1999 Fungi Europaei 7. Alassio: Mykoflora

[B45] Hesler LR Smith AH North American species of Lactarius 1979 Ann Arbor: University of Michigan Press

[B46] Romagnesi H Étude sur les lactaires de la sous-section des *Striatini* Bull Trimestriel Soc Mycol France 1974 90 139 146

[B47] Bon M Clé monographique du genre *Lactarius* Doc Mycol 1980 10 1 85

[B48] Nuytinck J Verbeken A Worlwide phylogeny of *Lactarius *section *Deliciosi *inferred from ITS and glyceraldehyde-3-phosphate dehydrogenase gene sequences Mycologia 2007 99 820 832 10.3852/mycologia.99.6.820 18333506

[B49] Brunner I Horak E Mycological analysis of *Alnus *associated macrofungi in the region of the Swiss National Park as recorded by J. Favre (1960) Mycol Helv 1990 4 111 139

[B50] Banaev EV Bazant V Study of natural hybridization between *Alnus incana *(L.) Moench, and *Alnus glutinosa *(L.) Gaertn J Forest Sci 2007 53 66 73

[B51] Roy BA Patterns of association between crucifers and their flower-mimic pathogens: host jumps are more common than coevolution or cospeciation Evolution 2007 55 41 53 10.1111/j.0014-3820.2001.tb01271.x 11263745

[B52] Grubisha LC Trappe JM Molina R Spatafora JW Biology of the ectomycorrhizal genus *Rhizopogon*. V. Phylogenetic relationships in the Boletales inferred from LSU rDNA sequences Mycologia 2001 93 82 89 10.2307/3761607 21156534

[B53] Hoeksema JD Thompson JN Geographic structure in a widespread plant-mycorrhizal interaction: pines and false-truffles J Evol Biol 2007 20 1148 1163 10.1111/j.1420-9101.2006.01287.x 17465924

[B54] Geml J Tulloss RE Laursen GA Sazanova NA Taylor DL Evidence for strong inter- and intracontinal phylogeographic structure in *Amanita muscaria*, a wind-dispersed ectomycorrhizal basidiomycete Mol Phylogen Evol 2008 48 694 701 10.1016/j.ympev.2008.04.029 18547823

[B55] Peintner U Bougher N Castellano MA Moncalvo JM Moser MM Trappe JM Vilgalys R Multiple origins of sequestrate fungi related to *Cortinarius *(Cortinariaceae) Amer J Bot 2001 88 2168 2179 10.2307/3558378 21669649

[B56] Boyle H Zimdars B Renker C Buscot F A molecular phylogeny of *Hebeloma *species from Europe Mycol Res 2006 110 369 380 10.1016/j.mycres.2005.11.015 16546367

[B57] Grubisha LC Trappe JM Molina R Spatafora JW Biology of the ectomycorrhizal genus *Rhizopogon *VI. Re-examination of infrageneric relationships inferred from phylogenetic analyses of internal transcribed spacer sequences Mycologia 2002 94 607 619 10.2307/3761712 21156534

[B58] Hedh J Samson P Erland S Tunlid A Multiple gene genealogies and species recognition in the ectomycorrhizal fungus *Paxillus involutus* Mycol Res 112 965 975 10.1016/j.mycres.2008.01.026 18554888

[B59] Gardes M Bruns T ITS primers with enhanced specificity for basidiomycetes - application to the identification of mycorrhizae and rusts Mol Ecol 1993 2 113 118 10.1111/j.1365-294X.1993.tb00005.x 8180733

[B60] Matheny PB Improving phylogenetic inference of mushrooms with RPB1 and RPB2 nucleotide sequences (*Inocybe*; Agaricales) Mol Phylogen Evol 2005 35 1 20 10.1016/j.ympev.2004.11.014 15737578

[B61] Johannesson HS Johannesson KHP Stenlid J Development of primer sets to amplify fragments of conserved genes for use in population studies of the fungus *Daldinia loculata* Mol Ecol 2000 9 375 378 10.1046/j.1365-294x.2000.00874-6.x 10736039

[B62] Jargeat P Martos F Carriconde F Moreau PA Gryta H Gardes M Phylogenetic species delimitation in ectomycorrhizal fungi and implications for barcoding: the case of the *Tricholoma scalpturatum *complex (Basidiomycota) Mol Ecol 2010 19 5216 5320 10.1111/j.1365-294X.2010.04863.x 21044190

[B63] Gonzales P Labarère J Sequence and secondary structure of the mitochondrial small-subunit rRNA V4, V6, and V9 domains reveal highly species-specific variations within the genus *Agrocybe* Appl Environm Microbiol 1998 64 4149 4160 10.1128/aem.64.11.4149-4160.1998 PMC106621 9797259

[B64] Mouhamadou B Carriconde F Gryta H Jargeat P Manzi S Gardes M Molecular evolution of mitochondrial ribosomal DNA in the fungal genus *Tricholoma*: barcoding implications Fungal Genet Biol 2008 45 1219 1226 10.1016/j.fgb.2008.06.006 18647655

[B65] Katoh K Kuma K Toh H Miyata T MAFFT version 5: improvement in accuracy of multiple sequence alignment Nucleic Acid Res 2005 33 511 518 10.1093/nar/gki198 15661851 PMC548345

[B66] Guindon S Gascuel O A simple, fast, and accurate algorithm to estimate large phylogenies by maximum likelihood Syst Biol 2003 52 696 704 10.1080/10635150390235520 14530136

[B67] Huelsenbeck JP Ronquist FR MrBayes: Bayesian inference of phylogeny Biometrics 2001 17 754 755 10.1093/bioinformatics/17.8.754 11524383

[B68] Posada D Crandall KA Modeltest: testing the model of DNA substitution Bioinformatics 1998 14 817 818 10.1093/bioinformatics/14.9.817 9918953

[B69] Posada D Crandall KA Selecting the Best-Fit Model of Nucleotide Substitution Syst Biol 2001 50 580 601 10.1080/106351501750435121 12116655

[B70] Swofford D PAUP*. Phylogenetic Analysis Using Parsimony (*and Other Methods) 2003 Sinauer Associates, Sunderland, Massachusetts Version 4d10

[B71] Felsenstein J Phylogenies from molecular sequences: inference and reliability Annual Rev Genet 1988 22 521 565 10.1146/annurev.ge.22.120188.002513 3071258

[B72] Drummond AJ Rambaut A BEAST: Bayesian evolutionary analysis by sampling trees BMC Evol Biol 2007 7 214 10.1186/1471-2148-7-214 17996036 PMC2247476

[B73] Drummond AJ Ho SYW Phillips MJ Rambaut A Relaxed phylogenetics and dating with confidence PLoS Biol 2006 4 699 710 10.1371/journal.pbio.0040088 PMC1395354 16683862

[B74] Rambaut A Drummond AJ Tracer v1.4 2007 http://beast.bio.ed.ac.uk/Tracer

[B75] Forest F Savolainen V Chase MW Lupia R Bruneau A Crane PR Teasing apart molecular- versus fossil-based error estimates when dating phylogenetic trees: a case study in the birch family (Betulaceae) Syst Bot 2005 30 118 133 10.1600/0363644053661850

